# In Vivo Bioassay of the Repellent Activity of Caraway Essential Oil against Green Peach Aphid

**DOI:** 10.3390/insects14110876

**Published:** 2023-11-14

**Authors:** Jessica Girardi, Kristīne Berķe-Ļubinska, Ieva Mežaka, Ilva Nakurte, Gundars Skudriņš, Laura Pastare

**Affiliations:** Institute for Environmental Solutions, “Lidlauks”, Priekuli Parish, LV-4126 Cesis, Latvia; kristine.lubinska@vri.lv (K.B.-Ļ.); ieva.mezaka@vri.lv (I.M.); ilva.nakurte@vri.lv (I.N.); gundars.skudrins@vri.lv (G.S.); laura.pastare@vri.lv (L.P.)

**Keywords:** *Carum carvi*, crop protection, essential oil, natural substances, repellent, *Myzus persicae*, integrated pest management

## Abstract

**Simple Summary:**

The green peach aphid (*Myzus persicae* (Sulzer)) is considered one of the main pests of economically valuable crops. In the last decades, it has developed resistance to several chemical pesticides. More effective and environmentally friendly solutions for green peach aphid management, including plant essential oils, are being tested as alternatives to chemical pesticides. Repellents have gained interest because lower doses can be used against agricultural pests, with a reduced risk of developing insect resistance. In this study, three different types of caraway (*Carum carvi* L.) seed essential oils against green peach aphids were tested. Repellent activity testing was carried out using a bridge method and host plants of white cabbage. The aphids were placed on the bridge, and their choice was recorded depending on whether they went to the treatment side (containing the selected essential oil) or the control side (no essential oil). All the selected caraway essential oils showed that they repel aphids to various degrees. The optimal essential oil dose was determined in the same way. In addition, the essential oil was mixed with different surfactants to create a formulation basis for ready-to-use products, but the testing showed decreased repellent activity of such mixtures.

**Abstract:**

An in vivo dual choice bioassay with white cabbage as a host plant was used to determine the repellent effect of three different accessions of caraway (*Carum carvi* L.) essential oils (EOs) against the green peach aphid *Myzus persicae* (Sulzer). The dominant components of the EO were D-Carvone (47.3–74.4%) and D-limonene (25.2–51.9%), which accounted for 99.2–99.5% of the EOs determined by GC/MS. The EO with the highest D-limonene content (51.9%) showed the highest repellence (Repellency Index (RI) = +41%), which was stable up to 330 min. The incorporation of several surfactants with different hydrophilic-lipophilic balance values (from 12.4 to 16.7) with caraway EO caused a general inhibition of the repellent effect during the testing period (RI from +41% to −19%). Overall, the findings indicate that caraway EO could be used as a green peach aphid repellent, but more work is needed to formulate the EO into a ready-to-use product.

## 1. Introduction

The green peach aphid *Myzus persicae* Sultzer (Hemiptera: Aphididae) is a well-known cosmopolitan pest both in greenhouses and in open-field environments [1]. It feeds on 50 plant families, installing and adapting, for example, on carrots, lettuce, spinach, cabbage, cucumber, beans, cereals, and different Solanaceae species [2].

**Figure 1:**
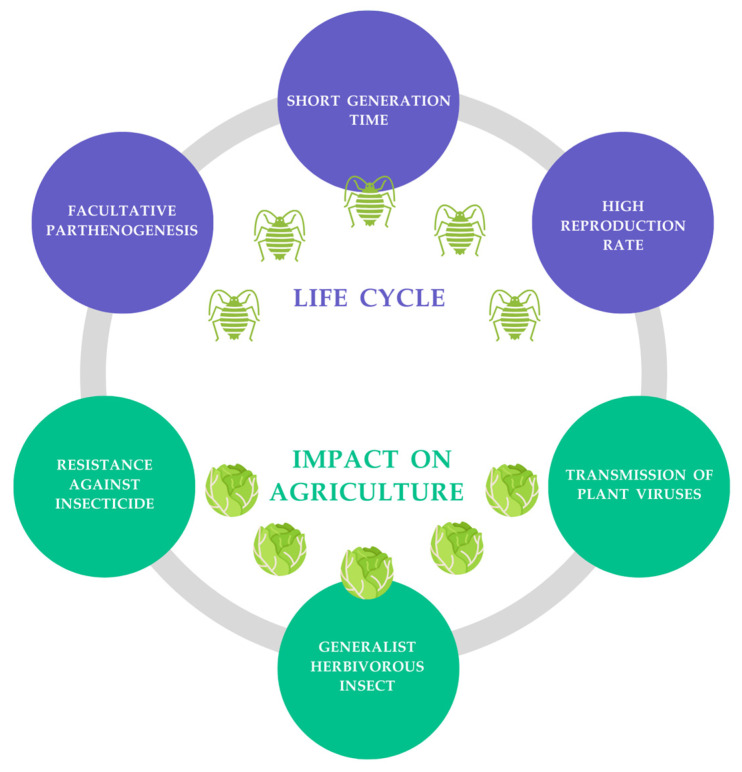
Factors that identify *Myzus persicae* as a cosmopolitan pest with high agricultural impact (modified from Ali et al. [2]).

The ecological success of *M. persicae* has been attributed to its short generation time and high reproductive potential, the ability to choose food sources from a wide spectrum of host plants, the transmission of severe viruses, and the development of resistance to synthetic insecticides (Figure 1) [2,3].

Olfaction plays a significant role in the pre and post-landing stages of host selection; in fact, aphids do not have much control over their movement, but they seem to respond to color and odor input [4,5]. Other than visual factors involved in host finding, plant volatiles (VOCs) are used by aphids to guide them to their host plants [5]. VOCs include two different categories of volatiles: green leaf volatiles, which are ubiquitous and do not carry information regarding the taxonomic identity of the source plant, and VOCs that are specific for a particular plant order, for example, the isothiocyanates that are emitted almost exclusively by Brassicales [6].

Synthetic pesticides sprayed on infested crops are conventionally used to manage green peach aphids [7]. Pesticides are now recognized for their controversial efficacy and effect, as insects can develop resistance to pesticides [8], pollute the environment [9], affect non-target beneficial organisms [7], and threaten human health [10].

Plant essential oils (EOs) are major candidates for plant-derived bioinsecticides that fit the sustainable biological standard of integrated pest management. The advantages of EOs are that they are biodegradable, proven to have low or no toxicity to mammals, and effective on specific targets without developing pest resistance due to their complex chemical composition [11,12,13]. Repellents have gained interest because lower doses than contact insecticides can be used against agricultural pests [14,15,16,17,18], and they are less likely to develop insecticide resistance [19].

Plant EOs are mainly obtained by steam distillation of the vegetative and generative parts of plants. The resulting oil is a complex mixture of volatile organic compounds, and their synergy usually shapes the biological properties of EO [13]. EOs are used in the food, cosmetic, and pharmaceutical industries. Their application in agriculture as insecticides, fungicides, and herbicides has been a topic of interest for several decades [20]. By definition, repellents are substances that act locally or at a distance, deterring a pest from flying to, landing on, or biting the target organism [21].

Many studies have investigated the effectiveness of EOs as contact insecticides, fumigants, and repellents against green peach aphids. Essential oils of medicinal and aromatic plants like *Foeniculum vulgare* [22], *Ocimum basilicum* [23], *Rosmarinus* officinalis [23], and many others [24,25] have been researched and proven to be successful insecticides in different testing settings.

The fumigant toxicity of *Foeniculum vulgare* EO and its major constituents has been researched against *Myzus persicae*. Research showed that a dose of 0.78–3.38 µL/L air can achieve 90% mortality in the population after 24-h exposure [26]. Research by Digilio et al. [27] showed green peach aphid mortality of 85% after 24 h exposure to *Carum carvi* EO dose of 2 µL/L air. The findings also highlighted that EO used at this dose has a phototoxic effect on the host plant (*Capsicum annuum*).

Several EOs have been studied regarding their repellence against green peach aphids. *Mentha pulegium* and *Origanum majorana* EO applied at 150 µL per test showed repellent activity of 80.5% and 57.5%, respectively, achieving the highest repellency at 150 min but decreasing afterward [28]. Similar dose-dependent repellency has been demonstrated with *Cotula cinerea* EO against green peach aphids, showing the repellency change from 48.5% to 64.6% to 37.4% by varying the applied dose from 70 µL to 150 µL to 300 µL [29]. The dose-dependent repellency has also been shown to occur over time: the same applied dose induces progressively different repellency responses [30].

Dried caraway (*C. carvi* L.) seed EO, whose principal components are D-Carvone and D-Limonene [31], has been researched as a fumigant and contact insecticide and as a repellent against stored grain pests, termites, and different aphid species. Carvone has shown repellent and insecticidal properties [32,33,34,35,36,37], as well as a bioherbicidal effect [38]. Limonene is reported to have insecticidal and repellent properties as well [39,40].

Caraway EO has been shown to have insecticidal activity against *Sitophilus zeamais* and *Tribolium castaneum* at doses 3.07 and 3.29 µg per insect, respectively, and fumigant activity at doses of 3.37 and 2.53 mg/L air. The EO had synergetic insecticidal and fumigant activity, as the use of the fractionated main compounds (D-carvone and D-limonene) had 10-fold weaker activity [41]. Caraway EO has demonstrated high repellency (91.7%) against *S. oryzae* at a dose of 10 µL in a T-tube bioassay. The research also revealed a similar trend as other studies: D-carvone and D-limonene alone have weaker repellency than EO, which consists of a mixture of several constituents [42]. Caraway EO 0.1% solution in ethanol has been shown to alter the feeding pattern of green peach aphids [43] and to repel them (41% repellency) [30].

The application of EOs for crop protection is currently limited by high volatility and low durability. The efficacy declines rapidly after EO application [13,44]. The main strategy to create an efficient delivery system with EOs is to use surfactants to create an emulsion or to encapsulate it [45]. The choice of surfactant affects the formation and performance of the emulsion. The contact toxicity of EO emulsions against various pests has shown higher mortality rates at lower applied doses [46,47,48,49] compared to non-formulated EOs. On the other hand, the fumigant toxicity of emulsions can be lower compared to non-formulated EOs [50,51]. This highlights the importance of performance testing throughout the formulation process of EOs into ready-to-use products, as it is a complex process that is specific for each EO and intended application.

Most studies investigating EO-repellent activity conduct tests in closed systems, such as Petri dishes [52,53,54,55] or olfactometers [42,56], where the behavior of the insect is observed in relation to the tested solution only, without plant VOC interaction. The addition of a host plant into the bioassay is important to evaluate whether the repellent effect is large enough to mask the host plant VOCs [57,58].

The aim of our study was to assess the repellent activity of caraway essential oils against the green peach aphid, *M. persicae*. The in vivo dual choice bioassay was conducted using white cabbage (*Brassica oleracea* var. *capitata*) as the host plant in the setting as the next experimental step to get closer to field conditions. Three accessions of caraway EO were tested, and the optimal dose of EO was determined. Phytochemical analysis of EO was carried out by gas chromatography—mass spectrometry (GC/MS) to link the chemical composition of the EOs to their repellent activity. The repellent activity of different surfactants added to EO was evaluated.

## 2. Materials and Methods

### 2.1. Plant Material

The plant material used in this study was dry *Carum carvi* L. (caraway) seeds, which were kindly provided by Field and Forest Ltd. (Priekuli, Latvia). Three different accessions of caraway, harvested in Priekuli parish, Latvia, in September 2022, were used. The seeds were harvested fully ripe and dried at 60 °C until the moisture content was less than 10%.

### 2.2. Chemicals

Ethanol (96%) was purchased from Kalsnavas elevators Ltd. (Jaunkalsnava, Latvia). The surfactants used in the testing were Polysorbate 20, Coco glucoside (BBFactory, Riga, Latvia), PolySol ^®^ PGA (SOCRI, Casottole, Italy), and Contact (AgroDan A/S, Brabrand, Denmark).

### 2.3. Essential Oil Distillation

Dry caraway seeds were used to obtain essential oil using a steam distillation apparatus, Albrigi Luigi SRL Dragon Pro 250 (Verona, Italy). A single distillation batch used 40 kg of dry caraway seeds (moisture < 10%), with a steam input of 23.8 kg/h, a condenser cooling capacity of 6 L/min, and a distillation duration of 2 h and 30 min. The essential oil yields determined for each caraway accession were 1.68% *v*/*w* (EO1), 1.35% *v*/*w* (EO2), and 3.2% *v*/*w* (EO3). The essential oils were stored at 4 °C in dark vials until further analysis and testing.

### 2.4. Essential Oil and Surfactant Mixture

The essential oil mixing was carried out with 96% ethanol and 4 different surfactants with varying hydrophilic–lipophilic balance (HLB) values from 12.4 to 16.7 (see Table 1 for the content of essential oil mixtures and surfactant descriptions). One EO that showed the best results in the first stage of in vivo testing was combined with surfactants at a ratio of 1:1 *w*/*w* (EO: additive), except for PolySol, where a ratio of 1:2 was used.

The mixture of essential oil and additive was agitated using a Witeg Overhead Stirrer HS-A (Wertheim, Germany) for 30 min at 10,000 RPM. The mixtures were tested for stability for 2 weeks at 54 °C. No visual, textural, or odor changes were observed in the tested mixtures.

### 2.5. GC/MS Analysis of Essential Oils

Caraway seed essential oil volatile constituents were determined and quantified using the GC/MS method in three replicates. Then, 500 µL of EOs were diluted with 500 μL of cyclohexane and mixed. Next, 50 µL of the obtained solution was diluted with 1450 μL of cyclohexane and analyzed using an Agilent Technologies 7820A gas chromatograph connected to Agilent 5977B mass selective detector equipment, featuring a Gerstel MPS autosampler (Mülheim, Germany). For separation, a polar CP-Wax 52CB capillary column (50 m × 0.32 mm, 0.20 µm film thickness) was used (Agilent Technologies, Santa Clara, CA, USA). Helium (He 6.0) served as the carrier gas, with a split ratio of 1:50 and a flow rate of 1.5 mL per minute. The temperature program was initiated at 60 °C and remained steady for 5 min before ascending at a rate of 3 °C per minute until it reached 250 °C. It was held at 250 °C for 3 min. The injector temperature was maintained at 250 °C. Mass spectra were recorded at 70 eV, spanning the mass range of *m*/*z* 50–500. The ion source temperature was kept at 230 °C. Identification of the components was based on their retention indices, determined in relation to a series of C5–C24 n-alkanes, and a comparison of their mass spectra with those stored in the National Institute of Standards and Technology MS search 2.2 library. The Agilent MassHunter Qualitative Analysis 10.0 data acquisition software was employed for the analysis of the GC-MS data (see Figure S1). Quantification of the separated compounds was accomplished by measuring peak areas using the normalization method without applying correction factors. The standard deviation (SD) was calculated for each detected compound.

### 2.6. Insect Rearing

Aphids *Myzus persicae* (Sultzer) (Hemiptera: Aphididae) were collected in the wild from naturally infested pesticide-free *Arabidobsis thaliana* and taxonomically identified at the Institute for Environmental Solutions (IES) Laboratory of Applied Entomology (Cēsis, Latvia). The colony from which the individuals were used in this study was reared for more than 2 years under laboratory conditions (16:8 L:D photoperiod; 25 ± 2 °C/20 ± 2 °C day/night temperature; 65 ± 5% RH) on white cabbage plants (*Brassica oleracea* var. *capitata,* hybrid ‘Storka’ F1 seeds from Takii Europe B.V., De Kwakel, The Netherlands). Host plants were grown indoors under controlled environmental conditions (22 ± 5 °C, 60 ± 20% RH). Insects were kept in 45 × 45 × 45 cm nylon mesh BugDorm cages (model No. BD44545 MegaView Science Co., Ltd., Taichung City, Taiwan). Ten-week-old plants were placed inside the cages as needed to replace the old ones, allowing adults to migrate to the new plants and have a constant supply of fresh food.

### 2.7. In Vivo Repellency Bioassay

A series of in vivo experiments on cabbage plants were conducted to evaluate the repellent activity of caraway EO against green peach aphids. A dual-choice bridge method protocol was developed based on Khaled-Gasmi et al. [59] with minor changes. The repellency bioassay was performed in 3 steps: repellence comparison of different EO accessions, optimal EO dose selection, and impact of surfactant addition to EO on repellent activity. Only the best options were tested in further steps to narrow down the number of bioassays.

**Figure 2:**
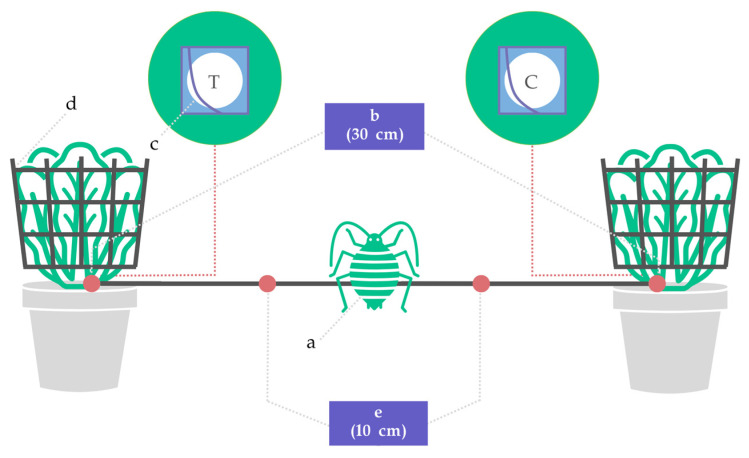
In vivo repellency bioassay of aphids on cabbage plants: (a) aphid releasing point, (b) plant distance, (c) filter paper disc in folder pocket placement (T = disc with test substance, C = control), (d) plant biomass supports, and (e) no choice zone.

Bioassays were carried out in 60 × 60 × 60 cm non-hermetic boxes with light dispersed from the top. A rigid inert plastic bridge with a rough surface (2.5 × 30 cm) was placed between two host plants, each containing treatment or control solutions on filter papers placed on the lowest plant axilla. Twenty-five aphids were positioned in the middle of the bridge, and their choice was recorded after 60 min (Figure 2).

Nine to ten-week-old white cabbage plants were used for testing. Ahead of testing, aluminum foil was used to cover the top-pot surface to eliminate any possible effect of soil volatiles. To harmonize the cabbage leaf area and avoid different visual stimuli that could affect insects’ choice [6], a plastic plant conic shape support, 13–16 cm in diameter and 15 cm in height was placed around the leaves, stabilizing them vertically.

Blank control (cabbage/cabbage) and control (cabbage with ethanol/cabbage, cabbage with surfactants/cabbage) tests were conducted to validate the bioassay method and assess the aphids’ reaction to ethanol (solvent) and surfactants. In the comparison of blank control, for both tested plants (Plant 1 and Plant 2), empty filter paper discs in folder pockets were placed on the lowest plant axilla. In the evaluation of the solvent effect, the treatment placed on Plant 1 consisted of 100 µL 96% ethanol, while an empty filter paper disc was placed on Plant 2. Finally, in the assessment of pure surfactant bioactivity, the treatment consisted of 100 µL surfactant, while the control was 100 µL 96% ethanol.

In comparing EO accession for repellence, the treatment consisted of a 100 µL solution of 50% *v*/*v* EO and 96% ethanol (50 µL of each), whereas the control was 100 µL 96% ethanol. The applied EO dose corresponds to 6.02 µL/cm^2^ on filter paper or 0. 23 µL/L in the experimental box.

In the selection of the EO dose for repellence, the treatment consisted of a 100 µL EO solution in ethanol, with the EO fraction ranging from 25% *v*/*v* to 100% *v*/*v* (25 µL to 100 µL, respectively), while the control was 100 µL 96% ethanol.

To determine the impact of additives on repellence, the treatment involved a 100–150 µL total solution, ensuring that the EO content was 50 µL in the applied volume of the solution. The control was 100 µL 96% ethanol.

Filter paper discs (2.3 cm in diameter) were chosen as the base for the application of the test or control solutions. A micropipette (Eppendorf Research plus, Hamburg, Germany) was used to apply solutions to the filer paper discs. Discs were left to dry for 30 min to allow full evaporation of the ethanol and then placed in 3 × 3 cm plastic (LPDE 4) folders with the front panels removed. Filter paper discs were finally placed on the lowest plant axilla.

In each replica, twenty-five apterous adult random-age aphids were placed in the middle of the bridge after a starving time of 4 h. To ensure that all insects were released at the same time, the bottom part of a 3 mL plastic syringe was cut, and aphids were gently introduced into the system using the syringe valve. The experiments were conducted in the same time frame (12:00–17:00) every day.

The number of insects on each plant (and corresponding bridge part) was observed 60 min from the insect-release moment. If the insect settled in the central area (5 cm from the release point, in both directions) of the bridge, it was considered a no choice.

After each test, the insects were gently removed from the system with a brush, and the bridge was replaced with a new one. To avoid the geographical position effect, the location of the cabbages was switched (180-degree rotation) after every test. The experiment was repeated 4 times, and in each replica, the results were obtained 90, 170, 250, and 330 min after substance application on a filter paper disc. For every substance, 16 replicas and 400 aphids were tested.

The repellency index (%) (RI) was calculated according to the following formula [60]:

$$RI,\%=\frac{\left(C-T\right)}{\left(C+T\right)}\times 100\%$$

where C is the total number of aphids on the control side, and T is the total number of aphids on the treatment side.

Positive and negative values indicate repellent and attractant effects, respectively. Referring to McDonald et al. [60], the substance was considered to have repellent properties if RI ≥ 0.1%, neutral if 0.1% > RI > −0.1%, while in the case RI ≤ −0.1%, the substance was considered attractant.

### 2.8. Statistical Analysis

Statistical analyses were performed using R v.4.2.2 (R Core Team, 2019). The choices of aphids were analyzed using generalized linear models (GLM) with a binomial distribution, with caraway EO accession or caraway EO concentration, or additive to EO as explanatory variables and the null hypothesis that an aphid would choose either side of the test with equal probability (0.5) [61]. Non-responding insects were not included in the analysis.

## 3. Results

### 3.1. Composition of Caraway Seed Essential Oils

Nine volatile components were detected in different accessions of caraway seed essential oils (Table 2). The dominant components were D-carvone (47.3–74.4%) and D-limonene (25.1–51.9%), which accounted for 99.2–99.5% of the EOs. The amount of minor components in all tested EOs was <0.8%. Oils had different EO profiles regarding carvone and limonene proportions. EO1 and EO2 had a higher carvone-to-limonene proportion, but EO3 had a similar proportion of limonene to carvone.

### 3.2. In Vivo Repellency Bioassay

**Figure 3:**
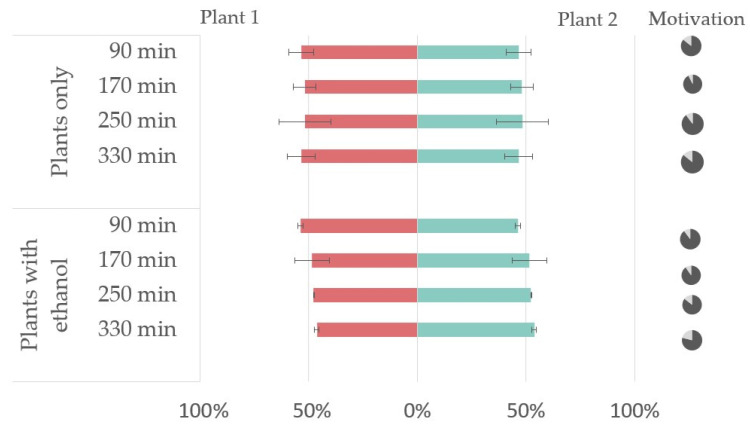
The results of the dual choice bioassay test of aphids choosing between two plants without any treatment (blank control) and between plants supplemented with a filter paper disc with 100 µL of 96% ethanol (Plant 1) or plants with an empty filter paper disc (Plant 2). The graph illustrates the percentage of insects choosing Plant 1 (red color) or Plant 2 (green color). The pie charts on the right represent choosing aphids (dark gray) and aphids not making choices (light gray).

Bioassay control tests were performed to validate the in vivo repellency using only cabbage (blank control) and cabbage with ethanol (control) according to the bioassay setup. The results of the blank control and control tests (Figure 3) showed no significant differences in the choice of aphids between the two directions (*p* > 0.05) and, thus, no bias within the experimental setup.

**Figure 4:**
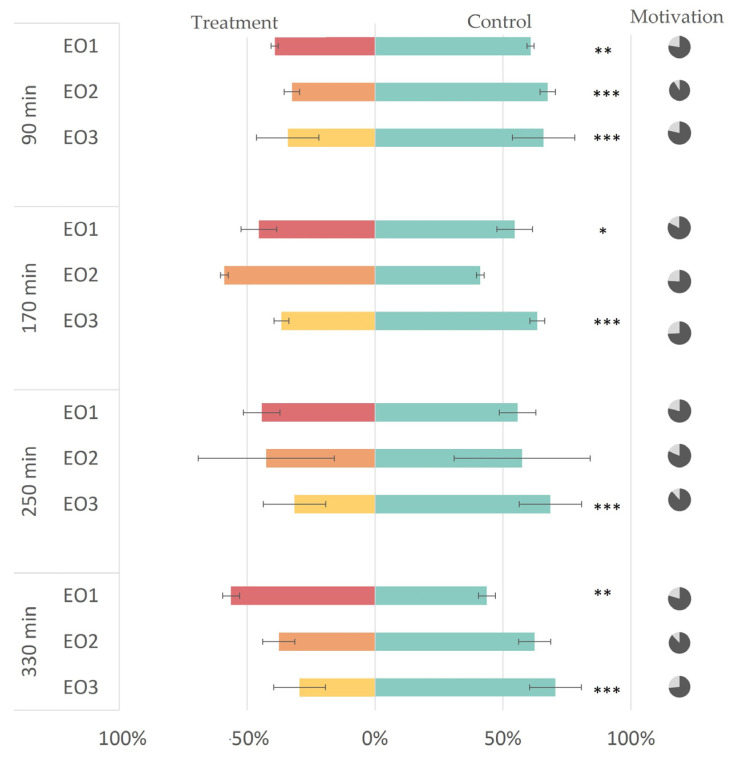
The results of the dual choice bioassay test of aphids choosing between the control plant (supplemented with a filter paper disc with 100 µL of 96% ethanol) or the treated plant (supplemented with a filter paper disc with 100 µL of 50% *v*/*v* EO in 96% ethanol solution). The graph illustrates the percentage of insects choosing the control (green color) or treatment (red, orange or yellow color for EO1, EO2 or EO3, respectively). The *p*-values of the generalized linear model (GLM) with a binomial distribution comparing insect preference to an equal distribution are represented by asterixis * (*p* < 0.05), ** (*p* < 0.01), and *** (*p* < 0.001). The pie charts on the right represent choosing aphids (dark gray) and aphids not making choices (light gray).

All three tested EOs (EO1–EO3) of different caraway accessions showed statistically significant repellency (*p* < 0.01) against *M*. *persicae* 90 min after application to paper discs (RI EO1 = +15%; RI EO2 = +35%; RI EO3 = +32%). However, throughout the experiment, it became evident that the repellent effect depended on the proportions of D-limonene and D-carvone in the EOs. The essential oil with similar D-limonene and D-carvone proportion (EO3) maintained the repellency over time, lasting until 330 min after the EO application to the filter paper disc, with a RI of +41%. In contrast, EOs with D-carvone as the dominant compound (EO1 and EO2) showed no statistically significant repellency after 250 min (RI EO1 = +5%; RI EO2 = +15%) (see Figure 4). The average number of aphids making no choice did not significantly differ among the three applied EOs (*p* > 0.05).

**Figure 5:**
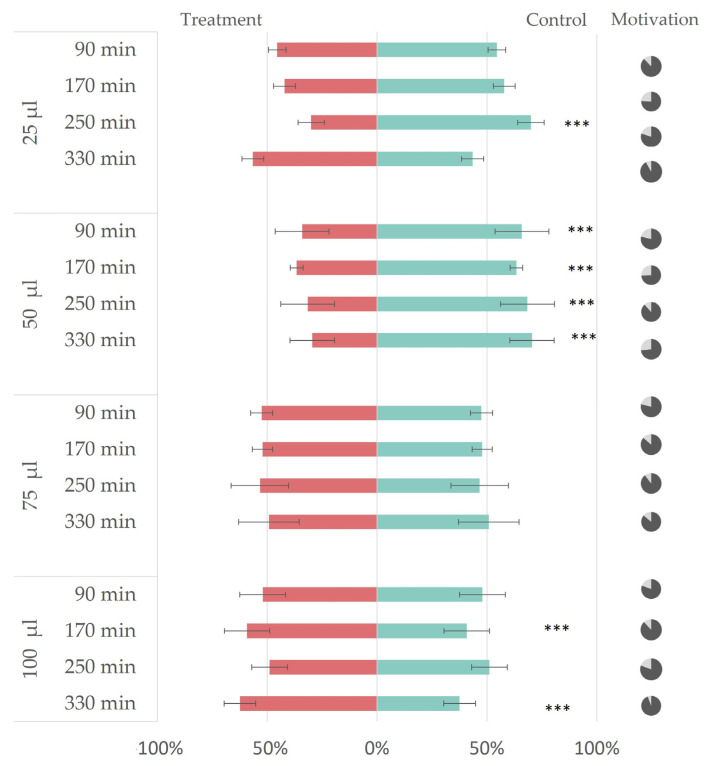
The results of the dual choice bioassay test of aphids choosing between either plant treated with a different amount (25–100 µL) of EO3 or the control plant treated with 100 µL of 96% ethanol. The graph illustrates the percentage of insects choosing the control (green color) or treatment (red color). The *p*-values have been determined by the generalized linear models (GLM) with a binomial distribution, comparing the tested distribution to a 50:50 distribution and comparing insect preference to an equal distribution, represented by asterixis *** (*p* < 0.001). The pie charts on the right represent choosing aphids (dark gray) and aphids not making choices (light gray).

Based on these results, EO3 was chosen for further testing of the applied EO dose impact on repellency. The amount of EO applied to the filter paper discs varied from 25% *v*/*v* to 100% *v*/*v* EO in 96% ethanol solution (25 to 100 µL of EO, respectively) and did show an effect on the insect choice. The 25% *v*/*v* and 100% *v*/*v* EO in 96% ethanol resulted in insects making choices without a statistically clear preference either for the control or treatment (min/max RI at 25 µL and 100 µL dose were −13%/+40% and −25%/+2%, respectively). In the case of 50% *v*/*v* EO in the 96% ethanol solution, insects avoided treated plants in a statistically significant way (min/max RI at 50µL dose were +27%/+41%), showing a clear response in terms of repellency throughout the experiment time (see Figure 5). According to the ANOVA, the average number of aphids making no choice did not significantly differ between the four tested concentrations (*p* > 0.05).

As the last step of testing, four different surfactants were mixed with pure EO3 (using the optimal EO concentration of 50 µL in bioassay), and their repellency was determined.

Aphid reaction to pure surfactants at 330 min is neutral for Polysol PGA and Coco glucoside. At the same time, Polysorbate 20 showed statistically significant attractivity (RI at 330 min = −20%), and Contact was statistically significantly repellent (RI at 330 min = +35%), meaning that different surfactants by themselves have different influence on the insect behavior.

**Figure 6:**
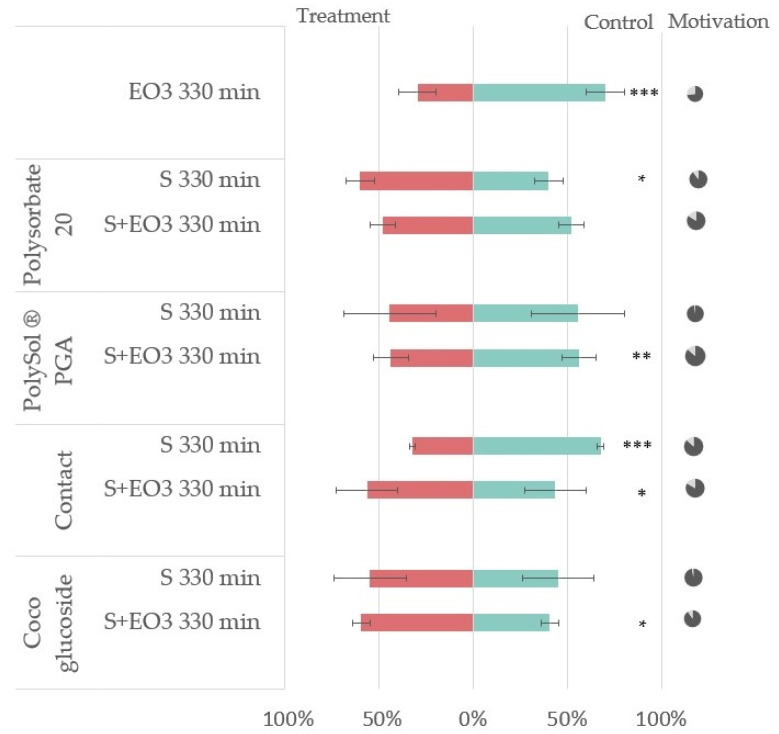
The results of the dual choice bioassay of aphids choosing between either plant treated with EO (EO3 dose 50 µL), surfactants (S dose 100 µL), or EO mixed with surfactants (S+EO3, where EO3 dose 50 µL), and the control plant treated with 100 µL of 96% ethanol. The graph illustrates the percentage of insects choosing the control (green color) or treatment (red color). The *p*-values of the generalized linear model (GLM) with a binomial distribution comparing insect preference to an equal distribution are represented by asterixis * (*p* < 0.05), ** (*p* < 0.01), and *** (*p* < 0.001). The pie charts on the right represent choosing aphids (dark gray) and aphids not making choices (light gray).

None of the tested surfactants in the mixtures with caraway EO showed comparable repellency to pure caraway EO at the end of the test (RI at 330 min = +41%). The combination of different surfactants with EO impacted the EO bioactivity in divergent ways: repellency, in case of PolySol PGA (RI at 330 min = +13%), neutrality, for Polysorbate 20 (RI at 330 min = +5%) and attractivity in the case of Contact and Coco glucoside (RI at 330 min = −20% and −19%, respectively) (Figure 6). EO3 results at the top of the graph are the same as in Figure 5 at 330 min and are reported here as a positive control.

## 4. Discussion

Terpenes, the major constituents of plant essential oils, play diverse roles in plant-insect relationships. In response to the environmental signals, the plants emit VOCs to repel herbivores and attract pollinators, parasitoids, and herbivore predators [62]. As a polyphagous insect with worldwide distribution, the green peach aphid must interact with numerous plant-emitted terpenes when seeking food, as they need to locate a suitable habitat, locate a host, and accept the host plant. External stimuli, including VOCs, guide these choices [63,64]. Several strategies have been developed to disturb host location acceptance, e.g., co-cultivation of crops with companion plants to disrupt insect behavior or application of plant extracts on crop plants [63].

In the present study, we demonstrated that the combination of caraway seed essential oil with cabbage plant VOCs exhibits repellence against green peach aphids. *Myzus persicae,* using in vitro tests with plant discs dipped in 0.1% caraway EO solution in ethanol, showed the highest repellency, +41% at the 2 h mark, decreasing to +35% after 24 h [30]. This aligns with our results, showing the highest demonstrated repellency of + 41% at 330 min. Both carvone and limonene, the major compounds of caraway EO, have been researched as compounds of interest regarding their fumigant and repellent activity. Seo et al. [65], demonstrated that both isomers of carvone have higher fumigant activity against *Reticulitermes speratus* Kolbe than both isomers of limonene. According to Giatropoulos et al. [66], repellent activity is isomer-dependent in limonene. Other researchers have also found that pure carvone has higher repellent and fumigant activity than pure limonene, but the combination of both compounds can achieve even higher activity against various pests [27,41,42]. This is also true for green peach aphids, whose response to individual compounds is not the same when compounds are mixed. It seems like aphids respond more to a combination of compounds than individual compounds [67,68]. In our study, higher repellency was achieved with EO containing similar proportions of carvone and limonene, while carvone-rich EO showed weaker repellent activity. As minor compounds in EOs also can have synergistic effects on the activity [42,69], they most likely also impact the results. Different clones or populations of *M. persicae* also can have different responses to VOCs [70], possibly due to morphological or physiological differences in the insect receptors: intermorph variation between apterous and alate aphids [67], odorant-binding proteins [71,72,73].

In our study, aphids showed dose-dependent repellency against caraway EO, with the highest repellency at a medium applied dose (50 µL per bioassay). Several authors have underlined that the activity of EOs in high concentrations is repellent and low concentrations—attractant [18,74,75,76,77,78]. Similar tendencies have been reported by other researchers [27,28,29,57], demonstrating that the same compounds or their mixtures (EOs) can be repellent at a peak concentration but attractant at lower or higher concentrations. The dose-dependent activity of EOs has been observed both in small-scale closed testing systems (petri dishes with filter paper or leaf discs), olfactometers, and open bioassays that include host plants and at various time points after EO application.

The bioassay results show significant attractivity of EO1 at 330 min and EO2 at 170 min while remaining neutral or repellent at other time points. A similar change in aphid response is registered with the used EO3 dose of 25 µL having increasing repellence up to 250 min and a sharp decrease to attractivity at 330 min (RI% at 90, 180, 250, 33 min +9%, +16%, +40%, −13% respectively). The tested dose of 100 µL also showed increased attractivity at 330 min (RI = −25%).

As the outlier response occurs at 330 min in several test runs, it suggests that at this time point, there is a change in the VOCs, influencing aphid responses. There are several possible reasons for the change in test box VOCs. Either the concentration or composition of the evaporated EO has changed, or the host plant emitted VOCs have changed.

It is a common practice to mention the whole applied EO amount (mass or volume) per bioassay or applied area or volume of bioassay, but that does not always correspond to the concentration of EO vapors. The evaporation rate of EOs and the EO vapor concentration in the air is dependent on many variables—temperature, moisture content, area of evaporation, dose of EO, duration, and EO composition, among others [79]. The changes in EO vapor concentration can also be caused by loss due to absorption, air exchange with the outside, and spontaneous decomposition or oxidation [80]. As most of these parameters were maintained at the same levels for the duration of the bioassay, it can be concluded that the impact of applied EO dose and composition on EO compound vapor concentration are the possible reasons for changes in aphid responses. For example, the oxidation of vapor phase D-limonene can occur within 30 min under specific conditions [80]. We suspect that at 330 min after EO application, the change in EO vapor concentration or composition could be large enough to evoke a different response from aphids.

A more likely source of changes in the VOCs in the test box is due to changes in host plant-emitted VOCs. In the bioassay, EO was applied on the filter paper placed close to the cabbage, not directly touching the plant tissue. The EO vapor concentration might be high enough to induce a stress reaction in the host plant. Volatile-mediated plant–plant interactions can cause stress reactions in the plant, leading to changes in their VOCs [81]. Early responses in plants to stress triggers can be observed as soon as 15 min after exposure but can depend on the plant, type of trigger, and other aspects [81,82]. The changes in plant-emitted VOCs can induce insect attraction [83] and could be responsible for the change in aphid response at 330 min.

The applied dose of EO can impact the aphid behavior and have a phototoxic effect on host plants. Studies on the fumigant effect of caraway EO against green peach aphids have demonstrated that doses (27 µL per test box or 2 µL/L) responsible for 85% mortality after 24 h exposure can also have a negative effect on host plants [27]. Scaling up the EO testing to the climatic chamber and greenhouse environment demonstrated a consistent effect on aphid mortality compared to lab testing but also revealed plant phytotoxicity [84]. A recent review published in the Annual Review of Entomology by Isman [85] claimed that a better demonstration and understanding of crop protection efficacy under field conditions is needed. One potential challenge associated with this approach is the possibility that the attractive effect of the plant, which serves as a food source, may outweigh the repellent properties of the substance [86]. Furthermore, it is necessary to clarify whether VOC emission modification due to biopesticide application [87] maintains the attractiveness of the plant as a food source. Baudry et al. [88] observed that despite *M. persicae* showing avoidance behavior towards leek (*Allium porrum*), the plant did not exhibit repellent properties when used as a companion plant in bioassay studies [88]. Results like that align more closely with field observations, making them particularly valuable to the industry [85,89]. Research must transition from proof of concept (traditional laboratory methods) to practical utility (in vivo bioassay) to meet industry production and commercialization demands [90].

In our study, we used a dual choice in vivo bioassay method to study the EO repellence effect on green peach aphids. The method is based in a laboratory setting but includes the pest host plant in the testing system to simulate a microenvironment closer to field conditions, where the EO and plant VOCs are mixed. A choice to extend the length of the bridge from 12–20 cm [59,86,91] to 30 cm was made with the intention of preventing plant contact and avoiding visual stimuli. The same setup can be used for evaluating sprayable formulations without additional adaptations. This step is advised for future studies as the plant extracts sprayed on crops modify the VOCs emitted [87]. Such changes in volatile composition can modify insect behavior [92].

An important step toward the commercialization of EO-based insecticides is their formulation into stable delivery systems. While there have been recent advances in the research of micro and nanoemulsions of caraway EO [93] and encapsulation of caraway EO [94,95,96,97], in most cases, the research lacks efficacy tests or comparisons with pure EO under the same testing conditions. Better nanoemulsion performance has been demonstrated in contact insecticide or larvicide testing, most likely due to the better penetration ability of the smaller EO particles in the emulsion [46,47,48,49].

Due to the reduced volatility and slower release of EO in emulsions or encapsulated solutions, their efficacy can be lower but more stable than that of pure EO [50,51,98]. Our results are in line with this, showing that the addition of surfactants to EO hindered the repellent effect, even in cases where the surfactant alone had a repellent effect (RI % for only EO3, only Contact and 1:1 mix of EO3 and Contact were +41%, +35% −20% respectively). These findings highlight the importance of continued bioassay testing during the formulation development process of EOs into ready-to-use products, as the concentration of EO in a formulation needs to be adjusted to achieve the same activity level as pure EO.

## 5. Conclusions

In this study, the repellent activity of three caraway seed essential oil (EO) accessions against green peach aphids (*Myzus persicae*) was evaluated in an in vivo setting, using white cabbage as the host plant. This study revealed that the repellent activity of caraway seed essential oil depends on the proportions of D-Carvone and D-Limonene. Specifically, the EO with a similar D-limonene and D-carvone proportion (53% and 46%, respectively) demonstrated higher and more stable in-time repellency at the applied dose of 50 µL EO. The repellency persisted over the course of the experiment (RI at 90, 170, 250, and 330 min was +32%, +27%, +32%, and +41% respectively). However, when different surfactants were incorporated with caraway EO, a consistent trend emerged: the addition of surfactants decreased the repellent effect of caraway EO. These results highlight the importance of ongoing repellent activity testing throughout the formulation process of EO-based repellent solutions. These findings highlight the significance of EO composition, additives, and dosage in the development of EO-based insecticides. This study underscores the potential of caraway seed essential oils as environmentally friendly solutions for managing green peach aphids in agriculture. Further research is essential to optimize formulations for practical agricultural use while considering potential phytotoxic effects in real-world scenarios.

## Figures and Tables

**Table 1:** Essential oil mixture content and surfactant characterization.

Commercial Name	Common Name	Manufacturer	HLB Value	Share in the Mixture
-	Ethanol, 96%	Kalsnavas elevators	-	50%
Polysorbate 20	Polyoxyethylene (20) sorbitan monolaurate	BBFactory	16.7	50%
Coco glucoside	C8–C14 fatty alcohol glucoside	BBFactory	13.5	50%
PolySol ^®^ PGA	Ester of Polyglyceryl-6 with Caprylic Acid and Proline	Socri	15	66%
Contact	Ethoxylated alcohols C9–11 > 90%	AgroDan	12.4	50%

**Table 2:** Volatile compound composition (%) ±SD (*n* = 3) in the essential oil distilled from caraway seeds.

No	Retention Indexes *	Compound	EO1	EO2	EO3
1	1118	β-Thujene	<LOD	<LOD	0.10 ± 0.00
2	1160	β-Myrcene	0.17 ± 0.01	0.19 ± 0.02	0.41 ± 0.05
3	1197	D-Limonene	25.12 ± 0.72	28.94 ± 0.91	51.89 ± 1.02
4	1593	Caryophyllene	<LOD	<LOD	0.13 ± 0.01
5	1617	Dihydrocarvone	<LOD	0.12 ± 0.02	<LOD
6	1624	trans-Dihydrocarvone	<LOD	0.12 ± 0.01	<LOD
7	1699	α-Terpineol	0.10 ± 0.00	<LOD	<LOD
8	1740	D-Carvone	74.38 ± 1.17	70.38 ± 1.00	47.33 ± 0.89
9	1792	Perylla aldehyde	0.24 ± 0.03	0.25 ± 0.02	0.13 ± 0.02

* Retention indexes determined on the CP-WAX capillary column, based on NIST (National Institute of Standards and Technology) MS Search 2.2 library; LOD—level of detection.

## Data Availability

The data presented in this study are available on request from the corresponding author.

## Supplementary Materials

The following supporting information can be downloaded at: https://www.mdpi.com/article/10.3390/insects14110876/s1, Figure S1: GC-MS mass spectra for all identified compounds in the tested Caraway seed essential oils, according to Table 2.
