# Supplementary material for: In Vivo Bioassay of the Repellent Activity of Caraway Essential Oil against Green Peach Aphid
Source: Insects. 2023 Nov 14;14(11):876. doi: 10.3390/insects14110876 (PMC10672326; doi:10.3390/insects14110876)
Supplement: Supplementary file 1 [file insects-14-00876-s001.zip › insects-2680191-supplementary.pdf]

Figure S1. GC-MS mass spectra for all identified compounds in the tested Caraway seed essential oils, according to Table 2.

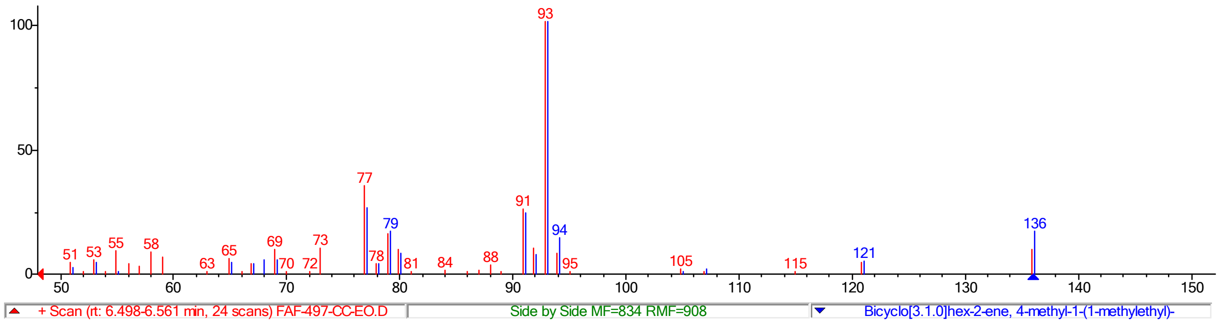

β-Thujene

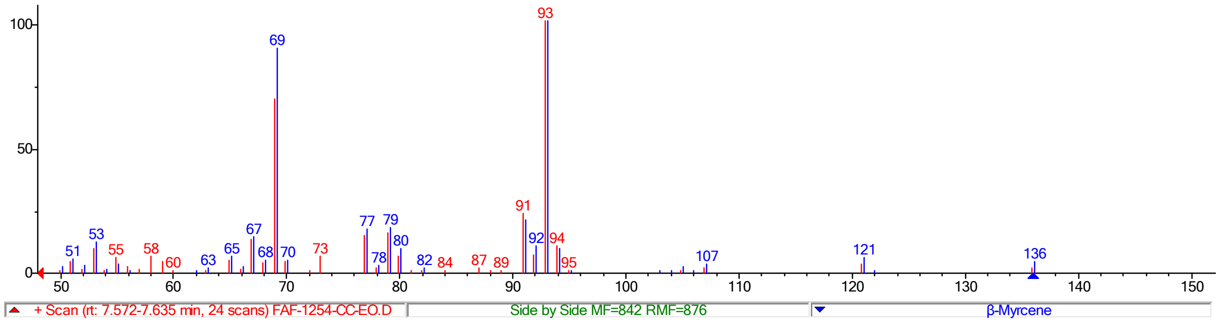

β-Myrcene

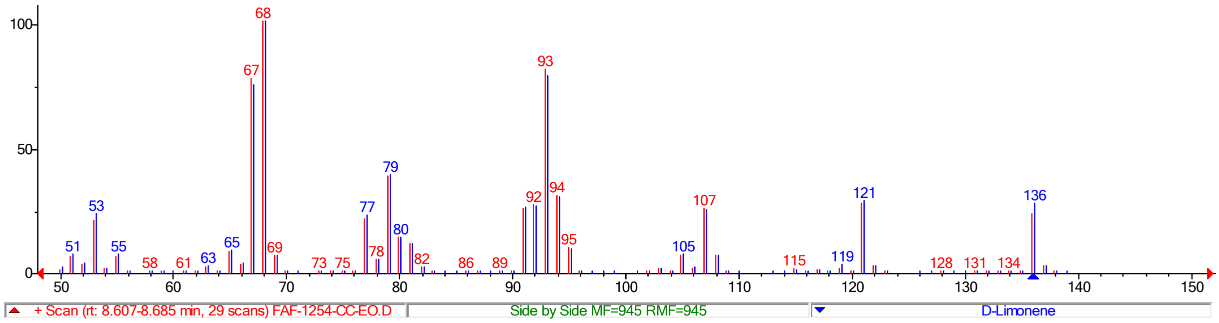

D-Limonene

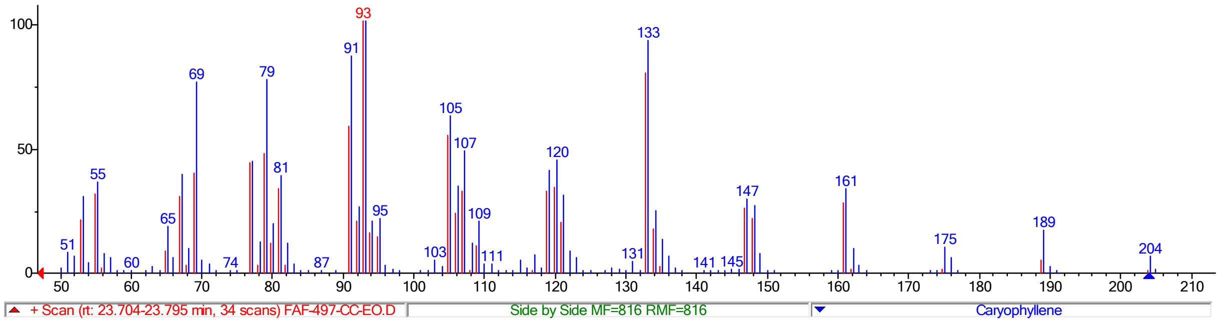

Caryophyllene

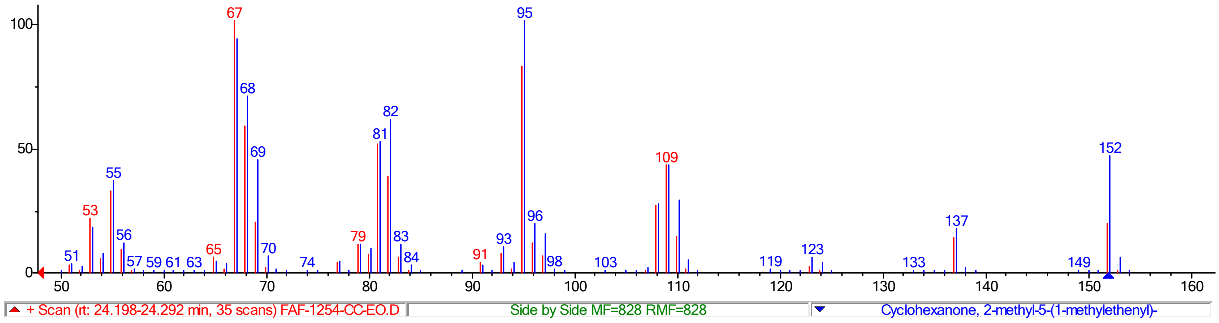

Dihydrocarvone

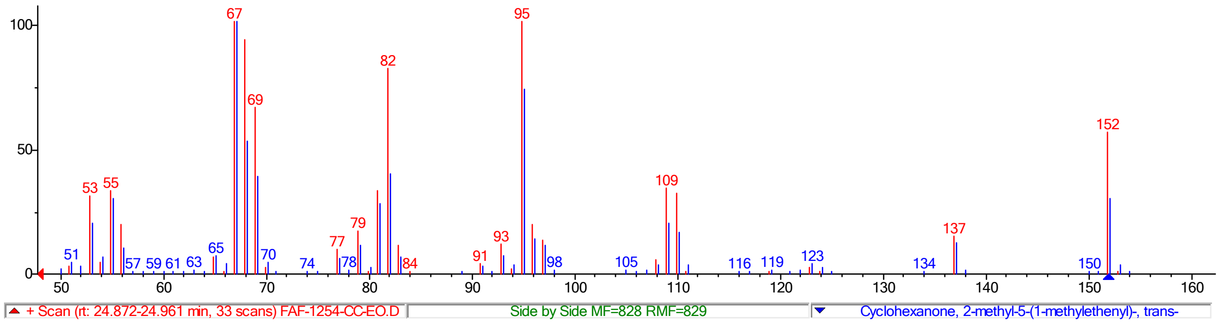

trans-Dihydrocarvone

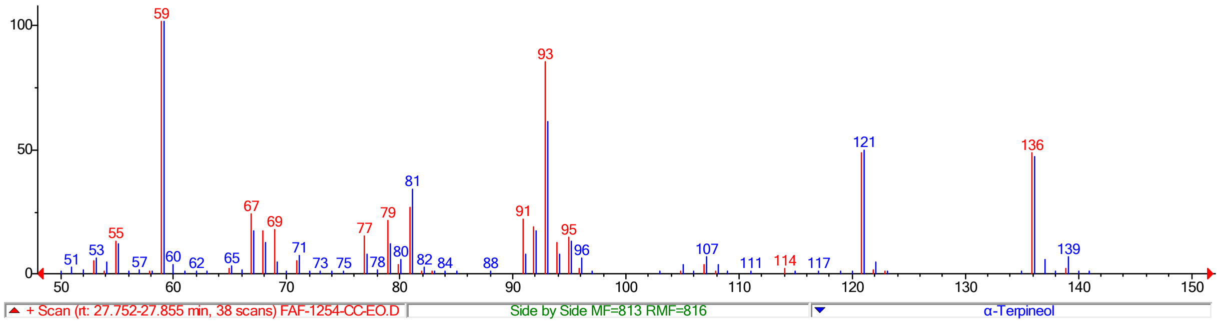

α-Terpineol

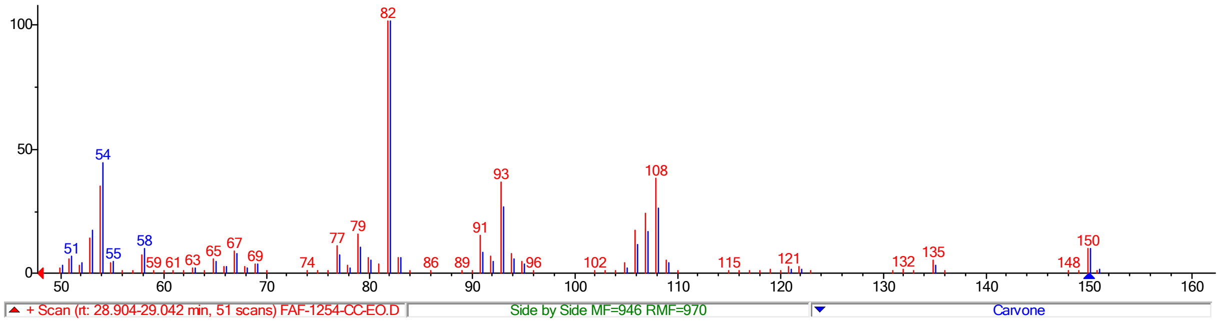

D-Carvone

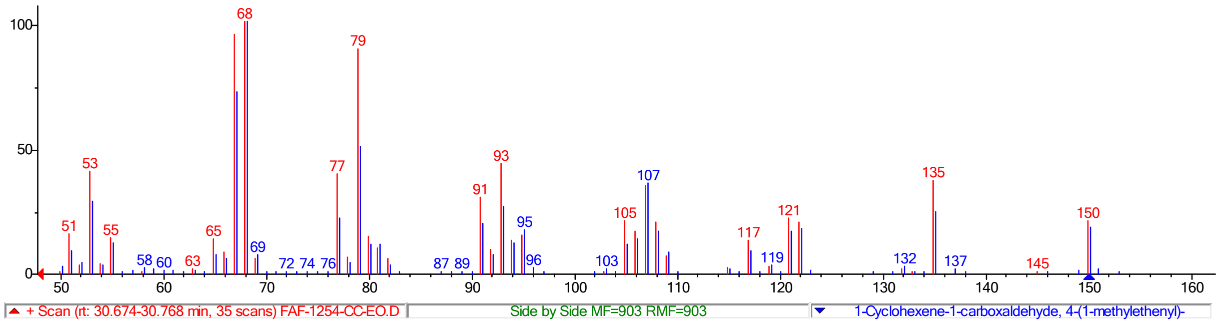

Perylla aldehyde
